# Transactions of the Chicago Gynæcological Society

**Published:** 1886-07

**Authors:** 

**Affiliations:** 2330 Indiana Ave.


TRANSACTIONS OF THE CHICAGO GYNÆCOLOGICAL SOCIETY.

*LXVI Regular Meeting, April 23rd, 1886.*

I.—CALDWELL. *Report of a Case of Hernia of the Umbilical Cord.*

II.—BARTLETT. 1.—*A Case of Dermoid Cyst Complicating Labour.*

2.—*A Case of Placenta Prœvia in which the Placenta was Expanded Over the Entire Ovum.*

The PRESIDENT, DANIEL T. NELSON, M. D., in the chair.

DR. CHARLES CALDWELL read a paper entitled,

REPORT OF A CASE OF HERNIA OF THE UMBILICAL CORD.

I was called August 10th, 1885, at 6 P. M., to attend Mrs. C., who was in labour with her ninth child. I made an examination and found a vertex presentation, first position ; os well dilated but no bag of waters had formed.

The first stage of labour was completed at 7 P. M., and the second an hour later. The child, a female, was quite large with very broad shoulders, which were difficult to deliver.

As soon as the child was expelled, I discovered she had a large umbilical hernia. I ligatured the cord and gave the child to the nurse, who removed it to another room, that the mother might not be alarmed at sight of the tumor.

The placenta was expelled by Credé's method and the uterus contracted firmly under pressure of the left hand.

After the binder was applied and the mother made comfortable. I examined the tumor more carefully. It was as large as a small orange and of similar shape. The circumference in the largest place,—the center,—was about ten inches. The diameter of the orifice or abdominal opening, two inches. The sac was translucent and the viscera,—intestines,—could be distinctly seen when the child cried and forced them out. The umbilical vessels were on the left side of the sac. The skin was projected from the abdomen on to the side of the sac at its base about half an inch.

*Diagnosis.*—Hernia of the umbilical cord.

*Pathology.*—Ectopia of some or all of the abdominal viscera at the point of insertion of the umbilical cord, is usually due to an arrest of development of the abdominal parietes, and failure of the intestines originally projecting into the vitelline duct to return into the abdominal cavity.

Simpson thinks it may sometimes be traced to peritonitis. Cleft abdominal walls, a similar arrest of development to that of cleft sternum, may exist in the muscular structure of the front of the abdomen, which may expose the whole contents of the abdomen, or the central line of the abdomen may be weak from deficiency of muscular structure.

If the orifice is very small, it can be treated successfully by a simple compress and adhesive straps. But if large, nothing except a plastic operation is indicated.

The coverings of the hernial sac are, from without inwards (*a*) amnion, (*b*) peritoneum.

*Treatment.*—After examining the sac thoroughly I was convinced that nothing but surgical interference could save the child's life.

I called our President and Dr. Jaggard by telephone, but both were too busy to assist me that night. They both recommended a cork compress held in position by adhesive strips, evidently thinking it an ordinary case of umbilical hernia.

I decided not to operate until the next day, and enveloped the sac with absorbent cotton, retaining it in place with a loose band.

The next afternoon, Drs. Dudley and Jaggard met me in consultation. After examining the little patient, they decided with me, that nothing but surgical interference would give her any chance of life, and kindly assisted me in the operation.

It was decided not to give an anæsthetic. Dr. Jaggard seizing the sac, gently forced its contents, the viscera, back into the abdominal cavity and I ligatured it as close to the abdomen as possible, but did not draw the ligature perfectly tight. At the suggestion of Dr. Dudley the sac was now opened, to be sure no portion or loop of the bowel was confined by the ligature.

The peritoneum being opened, the index finger was passed into the abdominal cavity to hold back the bowel and protect it from the points of the hare-lip pins, which were passed at right angles to each other through the narrow piece of skin at the base of the sac. Two more ligatures, carbolized silk, were applied beneath the pins and were drawn tight enough to close the opening. The outer layer of the sac was trimmed off leaving a stump one and a half inches long. Dr. Dudley passed a gathering suture round the stump sealing it more tightly. This completed the operation; after which the stump was dressed with iodoform,—a narrow strip of carbolized gauze passed beneath the pins,—several layers of absorbent cotton and a roller bandage. The child was restless that night, crying several times, but slept well the next day. I dressed the stump every day, reapplying a similar dressing. The bowels moved naturally until the 16th, when I discovered some fæcal matter on the dressing as I removed it.

On the 17th, all the fæces passed through the fistulous opening. The members of the family were very much alarmed and wished me to close the opening at once. I assured them that nature would close it without any further surgical interference.

I called on Dr. C. T. Parkes for his advice. After hearing the history of the case he said it would undoubtedly close by granulation in a few days.

For four days all the fæces passed by the fistulous opening, but on the fifth there was a little stain on the diaper. On the sixth more and on the seventh all the fæces passed *per rectum.*

The family were happy once more, for the fistula had closed. There were no symptoms of peritonitis at any time. Neither vomiting, tympanites nor pain. The patient did not cry more than babies generally do, but nursed and slept nicely, requiring no unusual care.

The bowel, at point of perforation, was held firmly bound to the abdominal walls by lymph, which was thrown out.

From this time the wound healed very rapidly. The end of the stump sloughed, leaving it quite short. The small intestine was probably the one perforated, for there was no odor to the discharges.

What caused the rupture of the bowel to take place I am unable to say. Probably, either a portion of the bowel was caught, and became strangulated by the ligature, or it was punctured by a pin, as that instrument was inserted.

Dr. Dudley says the surgical operation was certainly a laparotomy, for the abdominal cavity was opened and the index finger introduced.

DISCUSSION.

PROFESSOR DE LASKIE MILLER said he had seen numerous cases of hernia but thought this specimen was possibly more extreme than any he had seen.

THE PRESIDENT asked Professor Miller in how large a hernia he would deem operative procedure, of a plastic nature, necessary.

PROFESSOR MILLER did not think it possible to state, by actual measurement; so much depends upon the condition of the tissues surrounding the opening, that the absolute diameter of the opening would not be the governing principle. If the borders of the opening are of considerable thickness and the tissues well developed, it would be possible to reduce and probably cure a hernia independently of any cutting operation. He inferred that in the case described in the paper there was a deficiency of all the tissues except the peritoneum and the amnion, the skin projecting upon the sac one-half inch around the opening. He thought there was another explanation possible in this case, viz., that the duct leading from the umbilical vesicle remained or became patulous and the fæcal matter passed out through that; for the duct can be seen in the cord, even at delivery, in some cases. He inquired as to the cause of the arrest of development of the abdominal parietes in such a case.

DR. EDWARD WARREN SAWYER thought it not a very unusual thing to find a prolongation of the intestine into the cord. He once came very near tying the cord including a loop of intestine, so near that he had since observed the rule to always satisfy himself, by careful manipulation of the cord, that it contained no loop of intestine, and this routine practice had revealed the fact that very often a suspicious enlargement of the cord is found, sometimes extending three-fourths of an inch above the level of the abdominal wall and the intestine, or portion of the omentum, is not infrequently projected into this *cul de sàc*. He thought in the majority of cases nature would take care of the condition without any attention from the attendant. He had never seen so large a hernia as that described by Dr. Caldwell, and did not understand why it should have assumed a spherical shape; he could understand how it might be pyriform or sausage-shaped, and extend an inch or two into the cord, but how the cord could suddenly dilate and form a large globe, and this globe be followed with the contents of the abdomen, it was difficult to comprehend.

DR. SAWYER inquired what there was about this case that enabled the physicians to decide so quickly that an operation must be resorted to in order to save the child's life. To him an operation would have been a second consideration. Dr. Jaggard evidently reduced the hernia without difficulty, and having reduced it, it seemed to Dr. Sawyer that a well applied bandage would have secured it. The operation was brilliant and creditable, but he thought an operation should have been considered, after the attendants had failed utterly to secure the hernia within the cavity by a well applied bandage.

It was stated in reply to questions that the child presented no symptoms; the hernia was reduced by Professor Jaggard; the tumor about the size and shape of an orange; the operation was performed the day after birth; there was no evidence of strangulation of the gut.

DR. SAWYER said that Dr. Caldwell had assured him that he was certain that in passing the hair-lip pins no part of the intestine was included, and the finger was introduced to push everything beyond the reach of the pin. Dr. Sawyer thought, however, that the centrifugal pressure must have been considerable, and the contact quite severe. He thought it strange that none of the fæcal matter escaped into the peritoneal cavity, although there were adhesions between the intestine and abdominal parietes.

THE PRESIDENT: Of course every case must be decided on its merits, but might we not hope for the closure of the opening of an inch and a half in diameter, provided the tissues are well developed around it, without a cutting operation? It would seem possible to close as large an opening as in the case under discussion by simple adhesive strips. He had seen one case that reminded him of this: the opening was not nearly as large, it did not exceed three-fourths of an inch in diameter, and the length of the sac was much longer than the opening. Into this sac there was projected a solid body. As the child afterwards died, he found it was the *lobus Spigelii* of the liver. It was so conical, or heart-shaped, that the anatomy could not be made out until after death. The instruction to him in that case was the importance of not including any of the abdominal tissues by the ligature that might be passed around. He was careful to pass the ligature around the umbilical cord so as not to include the sac, but it might readily have been passed close to the abdomen and made to include the solid tissue, which, as he afterwards found, was a portion of the liver. The child died, not from the hernia, but from some want of development which he was unable to find out, no careful *post mortem* examination being allowed, but he believed there was lack of fœtal development necessary to life. The child was fairly well developed and seemed as if it should have lived, so far as this slight defect was concerned.

The PRESIDENT asked if any one could suggest the reason for the fæcal fistula. It seemed to him that in this case it was the result of inflammation caused either by pressure against the pins or the ligature; if the intestinal wall had been punctured by the pins, the physician would have seen evidences of it earlier.

PROFESSOR J. H. ETHERIDGE inquired whether this was not the youngest laparotomy on record. He wished to know if it would not be possible to crowd back the viscera and then hold them in place with adhesive strips.

PROFESSOR T. D. FITCH said that he had not had experience with congenital hernia at birth, but had seen it occur within twenty-four hours afterward, and had successfully closed an opening an inch or an inch and a quarter in diameter by the ordinary method, viz., compress and bandage.

Professor Fitch had had very peculiar success in the treatment of infantile umbilical hernia, by the ordinary means, and he should hesitate very long about an operation until he had tried all the ordinary means. His method of treatment differed somewhat from the ordinary treatment, however, in the adoption of an elastic web bandage instead of the ordinary bandage or adhesive plaster, and he thought it far superior to an inelastic bandage, as it gave the child room for an accumulation of intestinal gases, which very often become painful when a fixed bandage is placed around the abdomen. He first applied a hard compress, like a button-mole—(plano-convex)—to cover the opening. This should be large enough to extend three-eighths of an inch beyond the margin of the opening, and should be covered with one or two thicknesses of fine soft muslin. After returning the protruded substance *perfectly*, the compress should be fixed in position by short adhesive strips, then apply the elastic webbing around the body of the child tolerably tight and let each turn of the bandage lap the previous one half its width, making a sufficient number of turns to cover six or eight inches of the abdominal surface vertically. He would leave this on, undisturbed, unless the child became restless, or exhibited some indication of injury —for a month or longer before removing it. He had never found that the skin suffered from the confinement of the perspiration.

Another advantage was that the elastic webbing being rough and the laps of the bandage holding each other, it always stayed in place, and held the button firmly and so secured the opening against any possibility of protrusion. He had used this method successfully for about thirty years. He had a case at Waukegan which occurred twenty-four hours after birth; did not attend the mother at the birth, but was called to treat the hernia which was reducible, and would gurgle in and out at almost every respiration, bulging out as large as an English walnut.

He visited the child only once and told the parents to remove the bandage at the end of four weeks, when the opening was found entirely closed. The child is now nine or ten years old, perfectly healthy and always has been.

DR. CHARLES CALDWELL: The Fellows of the Society have evidently misunderstood the nature of the case described in the paper. I am sorry that such obscure and confused conceptions have been conveyed. The case was one of hernia of the umbilical cord, consisting in "the escape from the abdomen, at the point of insertion of the cord," of some of the fœtal abdominal viscera, and was due either "to arrested embryonic development, preventing the complete closure of the abdominal cavity, or failure of the fœtal intestines, originally situated outside the abdomen to enter the same" (Lusk). The remarks of most of the Fellows are accordingly irrelevant.

As no one who witnessed the operation was present at the discussion of my paper, several questions were unanswered.

DR. SAWYER wished to know why we decided so quickly that surgical interference was necessary to save the child's life?

The consultation was held about twenty hours after the birth of the child, and at that time the outer layer of the sac, the amnion, was dark and gangrenous in several spots. Its nutrition was cut off when the cord with its umbilical vessels was ligated, and it would have sloughed off in a few days leaving the viscera covered by the peritoneum only. We were of the opinion that such a condition as then existed would be followed by general peritonitis and death without some surgical operation.

DR. BYFORD wished to know the literature of the subject. I have been able to find but one case similar to mine.

Thomas Bryant, in the last edition of his "Surgery," mentions the only case he ever saw and his treatment. In June, 1876, a child, one day old, was brought to him with hernia of the cord. The sac was translucent, the size of a small egg, and contained the cæcum and vermiform appendix. He pressed back the bowel with the thumb and forefinger, stitched up the cord at the umbilical orifice with deep sutures and ligatured the cord itself at the apex of the congenital hernial sac.

Recovery was complete without a single bad symptom. He recommends his operation in all similar cases, evidently considering it the only treatment indicated. So we were supported by the best of authority, in operating instead of trying to apply a compress. Should I ever meet with a similar case, I would perform an operation different from either Bryant's or mine.

I would first remove the amniotic layer of the sac, if it could be separated from the peritoneum, excising or amputating it at its junction with the skin, return the viscera and peritoneal sac to the abdominal cavity and close the abdominal opening as in a case of exploratory incision, or simple laparotomy. Either incising the peritoneal sac to better protect the bowels from needle points, or stitch to the bottom of the wound by deep sutures, and support the sutures by adhesive straps around the abdomen.

I would recommend this operation after observing how quickly the amnion sloughed away. It might just as well be removed at once if it can be easily separated from the peritoneum.

DR. JOHN BARTLETT made remarks upon, (with the exhibition of specimens of)

(1)—A CASE OF DERMOID CYST COMPLICATING LABOR.

(2)—A CASE OF PLACENTA PRÆVIA IN WHICH THE PLACENTA WAS EXPANDED OVER THE ENTIRE OVUM.

Dr. Bartlett said, "I have recently seen two interesting cases of labor, and I wish to present a specimen obtained at each of these, to the SOCIETY.

The first was a case in which I was called to assist Dr. John S. Clark. The patient, a primipara, about thirty years old, had been in labor under the care of a midwife about twenty-four hours. The head was making no progress, and exhaustion was approaching; about one ounce of fluid extract of ergot had been given. Dr. Clark found the head lying with the antero-posterior diameter corresponding with the conjugate, the parietal eminences had passed the brim. He applied an old style, high curve, Bedford's forceps, but found his efforts unavailing in causing the head to advance. Dr. Bartlett then attached his direct traction handle, and descent of the head was effected. After delivery of the head there was difficulty in delivering the shoulders. When an effort at extrusion was made, there appeared in the perineal region, between the vulva and tuberosity of the ischium, a jutting outward of the tissues in the form of a tumor. It seemed as if an obstructing body was wedged in front of the shoulders. Counter pressure was made upon the protrusion, and the delivery was completed. Following the child, came a tumor of the shape and size of a large pear, presenting at the small extremity a pedicle. It was a thin dermoid cyst containing a mass of fatty substance, embedding numberless long intertwining hairs. The tumor could not be felt during labor, and a careful inspection showed that it had not been attached to the child. It was probably attached to the uterine surface, resting between the head and the shoulders. Possibly it was the cause of the dystocia in diverting the head from an oblique diameter of the brim. Depression of the vital powers with high fever set in soon after delivery, resulting in death within four days. Dr. Clark's examinations *post partum*, detected no injuries beyond laceration of the perineum. The child was still-born.

The second case was one of *placenta prævia.*

Mrs. N. had had several children, and within eighteen months past, two miscarriages. In January last, when she was nearly four months advanced in pregnancy, I was called because of serious hæmorrhage. The uterus presented to the touch nothing peculiar, there was none of that extra development of the lower segment of the organ which is supposed by some to indicate *placenta prævia.* The tampon was applied, opiates given, rest enjoined, and the bleeding ceased. At four and one-half months the hæmorrhage recurred. Under treatment, the bleeding was in some measure controlled. For the two following months it was continuous, generally moderate, occasionally quite severe, at all times, as she declared, four-fold greater than the flow of menstruation. I then deemed it best at six and one-half months to induce labor, but Dr. John S. Clark, in consideration of the probable non-viability of the child, advised further delay. The flow, however, was so great that the tampon was applied, and in forty-eight hours thereafter labour began.

Upon removing the plug, the os was found thin, softened, and three-quarters of an inch in diameter. At about the fourth hour of labour, the os rather suddenly enlarged to a diameter of one and a half inches, and the hæmorrhage became profuse. The half hand was introduced into the vagina, and the placenta stripped off over an area, the radius of which was three-fourths the length of the middle finger. With the bullet forceps, used by me with advantage in such cases, the membranes were torn, and the opening so made was freely enlarged by the finger. Hæmorrhage immediately ceased, and labor became more active, so that in the course of an hour the child was delivered, breech first. Several inspirations were made by the fœtus, a fact of interest in view of the peculiarities of the placenta, which I here present. In order to display the specimen to better advantage I have filled the cavity of the membranes with horse hair and sewed up the aperture. It will be observed that the main body of the placenta, the normal placental mass, is not *prævia*, but attached near the fundus, and that the rare anomaly is here presented of a continuous placental tissue spreading over the entire ovum. Observe that the extra, adventitious portion, continuous with the normal placental edges, and everywhere enveloping the membranes, is comparatively thin; in the present state not thicker than one-eighth or three-sixteenths of an inch.

PROFESSOR W. H. BYFORD spoke of a specimen he exhibited some time ago at the Chicago Medical Society—a dermoid cyst which was expelled from the vagina. It was sent to him by Dr. White, of Bloomington, who said the tumor was situated in the anterior wall of the vagina, and as the child was delivered the pressure of its head pushed the tumor out before it. He thought that in the case under discussion the tumor may have been developed in the vaginal walls. The localities of these growths are not uniform and we find dermoid cysts situated in the vaginal walls. Dr. Bartlett said in answer to a question from Professor Byford that the existence of the cyst was not discovered before but during labour; that it was beyond the head and may have been an ovarian tumor; it was not outside the vagina, but between the head and shoulders. He thought it almost certainly a dermoid tumor of the vagina.

PROFESSOR W. H. BYFORD thought that these cases are almost always found in old or multiparous patients.

With reference to Dr. Bartlett's second case, he asked if the hæmorrhage ceased before rupturing the membranes, or if the whole operation was done at once. Barnes claims that if the membranes are separated over the cervical zone the hæmorrhage will stop; that there will be sufficient retraction of the cervical zone to close up the mouths of the vessels. He thought it a point of interest to know whether or not that would have stopped the hæmorrhage, and whether it would not have been sufficient. He thought in that case one is not called upon to leave the membranes intact.

PROFESSOR DELASKIE MILLER said that the effect of endometritis is usually to increase the area of the development of the placenta, and he had not infrequently seen cases of placenta of the usual size, on which projections appeared in different parts in the interior of the uterus, partially connected or entirely disconnected. He asked whether a case of endometritis might not allow the villi of the chorion to form these *placentæ succenturiatæ.* He was inclined to think that condition would encourage it. Another fact in the history of the case that would perhaps justify this theory was the several miscarriages the patient had experienced before this pregnancy.

PROFESSOR T. D. FITCH said that in thirty-five years of practice, in which he had probably attended more than a thousand cases, he had seen but one case in which he suspected *placenta prævia*. He was sometimes ashamed to make the statement for fear his experience had been from lack of close observation, or inability to recognize a case, but he had seen only one, and did not know that that was really a case of *placenta prævia*. He did not detect it by manual examination; the symptoms were altogether subjective. It was a seven months' labour and the child lived. There was a good deal of hæmorrhage. He had no difficulty with the labour, except from the hæmorrhage, and that did not prove serious.

THE PRESIDENT asked if any one could suggest the origin of the tumor? Was it a twin or a dermoid thrown off from the fœtus, or was it from the mother. He asked Dr. Bartlett if it was a part of the child, and was answered that after careful examination no place was found where it might have been attached. It was perfectly loose in the vagina; probably the pedicle had been ruptured.

DR. EDWARD WARREN SAWYER thought (with reference to the second case) the case had several very interesting features, and the subject itself is full of interest. He referred to a conversation earlier in the evening in which Professor Byford had said that he had been in practice many years before seeing a case of *placenta prævia*, and the first case he ever saw was the first of three in one night. It had happened to Dr. Sawyer to see a number of cases of *placenta prævia*. He had had two fatal cases and had learned something of early diagnosis, which had been profitable to him since, and he thought of it in the obscurity of the diagnosis in Dr. Bartlett's case. He said that in one of his cases, after reading of the ease with which one could auscultate the lower segment of the uterus under these circumstances, he prolonged his stethoscope with a long flexible tube, put a cup on the end of it and had no trouble in introducing it into the woman's vagina. He had repeatedly detected portions of the cervical attachment of the placenta by this mode of auscultation. The remark made by Professor Byford had received some confirmation in his experience, viz., that many cases of *placenta prævia* aborted early and that he believed this to be a frequent cause of early abortion. Spiegelberg says that *placenta prævia* is of very frequent occurrence. An interesting fact in connection with Dr. Bartlett's case was the alarming amount of hæmorrhage which took place from the placenta and placental attachment away from the main part of the placenta, or in other words, there were sinuses in the cervical zone of this uterus which were covered only by the velamentous portion of the placenta, sinuses large enough to bleed, and exsanguinate the woman. Dr. Sawyer spoke of a case to which a former pupil of his was called. A midwife summoned him at midnight to see a woman who was bleeding to death. As he entered the room she handed the doctor a cord (the child was delivered), and the placental end of the cord was a disk about as large as a butter dish. That fleshy mass had been pulled directly from the placenta and the woman was actually bleeding to death. This little mass was very thin and he was at a loss to understand to what part of the placenta it could have been attached He delivered the woman completely and she was saved. He found a hole through the placenta corresponding to the disc which had been pulled out, it was in the thin portion of the placenta, and the bleeding was somewhat alarming. Referring to the question that Professor W. H. Byford asked concerning the mode of treatment advocated by Barnes, Dr. Sawyer said he was full of the idea, and the feasibility of it was accepted by him in his first reading of Barnes' work, and he tried to adopt it in practice, but he hoped no one would ever get himself into such a dilemma, as he was sure he lost his patient by that course. Theoretically you may detach enough of the placenta to save the woman from hæmorrhage. The so-called cervical zone is not to be measured by the finger, he did not think it had any definite boundaries; it might sometimes extend half way to the fundus on one side of the uterus and he thought the more we detach the more dangerous it may become. He felt quite confident that the poor woman who was the victim of the theory of Barnes would not be dead to-day if he had adopted a more rational treatment.

There was one point in connection with the causation of *placenta prævia* that excited a great deal of interest in his mind. He had seen two cases strangely confirmatory of the movement of the ovum in the early days of its sojourn in the uterus. One case was also seen by the PRESIDENT, but he had never spoken to him of the theory which the examination of the placenta had been the origin of. Dr. Sawyer had written to several prominent obstetricians of this country, asking if they knew anything in the literature which would answer the question: Can the ovum once attached to the decidua of the uterus become detached and again attach itself to the lower part of the uterus and go on through pregnancy? In other words, can the ovum detach itself, drop from the top of the uterus to the bottom, re-attach itself in the cervical region. Many learned men replied that they had never heard of such a possibility. But Dr. Harris, of Philadelphia, hit upon this happy expression: "Rotation of the ovum." He had seen two cases in which he thought the ovum rotated in the earliest days of pregnancy; not a complete rotation, but an almost complete detachment and rolling, or rotating, downward and there attaching itself. The second case was in the practice of Dr. Doering. The subsequent examination of the placenta showed a case of partial *placenta prævia.* The umbilical cord springs from the margin of the placenta in both instances; and his theory was that in the first case, which was that of a young primipara, the placenta was fixed normally at the fundus and the cord sprang from the middle; there was a history of a sudden jar of the body when she was about three weeks pregnant; she jumped from a high wagon and immediately flowed a little, and when she came to be delivered she had a complete *placenta prævia.* The examination of the placenta showed that the cord was attached to one margin, showing that it had rolled down and formed a new attachment for the umbilical cord. When the placenta was at the fundus and the cord at the middle, by its rolling downward it placed the cord at the margin. He thought one of the most important causes of *placenta prævia* is this rotation of the ovum very early in pregnancy; a rotation that may be caused by a sudden jar, and he thought the confirmation of it is in the unusual attachment of the cord.

PROFESSOR J. H. ETHERIDGE had seen one case of a woman with *placenta prævia* with twins. At about the fourth month the woman fell and struck the lower part of the abdomen against the top of a wash tub, she had a little hæmorrhage, and from that time till term she was always tender at that spot. Free hæmorrhage took place and he was called to see her. The woman was delivered of a mature fœtus, and of a child that was evidently arrested in development about the fourth month. The theory formed was that there was a partial detachment of the second placenta, the other child went on and developed regularly. The mother died in a few hours after delivery.

THE PRESIDENT wished to say a word about the theory of causation advanced by the SECRETARY, the idea of displacement, or rotation of the ovum after implantation. It seemed to him that there was a more important cause, and in all the cases he had personally investigated, about four, there had apparently been good reason for the theory that disease of the uterine mucous membrane, inflammatory and with an unusual pathological amount of secretion over its surface, is the cause of the implantation at the cervix instead of at the normal place, in the vicinity of the entrance to the Fallopian tube. From the quantity of the mucus the ovum glides down to the cervix and remains there, because the tissues are more healthy, perhaps. In two cases of *placenta prævia* that he had knowledge of pregnancy occurred some time after treatment, with a previous history of sterility, or of miscarriages, and, possibly the mucous membrane nearest the cervix was in a more healthy condition than that near the Fallopian tubes, or possibly there was constriction of the internal os. It seemed to him there was good reason for the belief that if the mucous membrane at the fundus is not in a good condition to nourish the ovum and to hold it, it falls to the internal os and is there held, and there is a possible *placenta prævia* at full term. He wished to know if in Professor Etheridge's case there was any previous knowledge of uterine disease. Professor Etheridge replied that there was none, the woman was a healthy Scotch woman.

PROFESSOR ETHERIDGE asked whether *placenta prævia* is a common thing in animals.

DR. SAWYER replied that he had seen a mare throw off her placenta before she threw her colt, and in fact she died without throwing the colt. He said further that he believed cervical pregnancy is generally recognized as being secondary to another thrown off from the fundus; that being the case, why not a secondary attachment or lodgment of the ovum at the lower segment of the uterus, as well as in the cervix itself. He believed the majority of authorities is against the idea of cervical pregnancy in the abstract, but he thought Dr. Bartlett considered it possible to conceive in the cervix.

THE PRESIDENT said there might be disease of the uterus there, and then comes the question whether that is the sole cause, or one of the many causes. The question had been asked whether *placenta prævia* is of frequent occurrence among prostitutes, women who might be supposed to be anxious not to become pregnant, and he thought it had been answered in the negative.

DR. SAWYER asked if the PRESIDENT'S theory was correct and his observations had been confirmatory of it, how is it that Spiegelberg can say that many primiparæ, young, healthy women miscarry on account of *placenta prævia*.

DR. SAWYER thought that pregnancy itself was infrequent among prostitutes.

DR. H. P. NEWMAN mentioned a rare case which had lately come to his notice, namely, a complete central implantation of the placenta, in which no hæmorrhage had occurred throughout the entire pregnancy until the very last days of gestation.

Ten days prior to delivery at full term there was the first appearance of bleeding,—easily checked by assuming the recumbent posture; and it was not until five days later that the hæmorrhage became at all abundant.

Delivery took place on Tuesday, April 13th.

On the preceding Friday Dr. R. N. Hall was called, and diagnosed *placenta prævia*, using the tampon.

I first saw the case in consultation with Dr. Hall on Tuesday morning.

The repeated tamponing and use of the colpeurynter the night before, had had the effect of gradually bringing on labor pains, and softening and dilating the cervix to the diameter of nearly two inches.

A digital examination revealed nothing but a thick placental surface upon all sides, covering, as we afterwards found, the entire lower segment of the uterus.

By bimanual palpation we made out a shoulder presentation (left dorso-anterior), and decided on immediate delivery.

Every preparation being made to control hæmorrhage, the placenta was carefully separated from its uterine attachments upon the left side, and the right hand carried upward between the membranes and uterine walls.

When the feet were reached the sac was ruptured, podalic version performed, and the child extracted.

Meanwhile Dr. Hall had followed up the evacuation of the uterus by firm bimanual pressure upon the uterus through the abdominal walls.

The placenta, which was a large one, and pretty evenly distributed upon all sides, was separated from its remaining attachments, and removed as speedily as possible.

The entire procedure was accomplished in less than five minutes, and the hæmorrhage was not excessive considering the gravity of the situation.

The child was saved, and notwithstanding the amount of blood lost by the mother at and previous to delivery, she convalesced rapidly, and is now up and about.

She is a strong, healthy woman of middle age, has borne seven children, and has had three miscarriages.

With the exception of rapid childbearing, a laceration of cervix, and one faulty presentation necessitating version, her former history has no particular interest.

In closing the discussion DR. BARTLETT said, that in a paper written years ago he had expressed an opinion that *placenta prævia* was one of the simplest of the *errores loci* of the ovum, that the true site of the placenta, when *prævia*, was the cavity of the cervix, that is, below the *os internum*, or the so-called ring of Bandl. Dr. Bartlett took occasion to emphasize his conviction of the truth of the position taken by him in the paper referred to. In the case now before the SOCIETY corroboration of his idea might be found. An ovum resting in the comparatively large cavity of the uterus would take root on the surface to which it chanced to be more nearly apposed. An ovum arrested in the much more circumscribed cavity of the neck might secure a more general, and in some rare instances, a complete attachment to surrounding uterine tissues.

W. W. JAGGARD, M. D., *Editor*.

2330 Indiana Ave.

14th June, 1886.

